# Medical insurance policy organized by Chinese government and the health inequity of the elderly: longitudinal comparison based on effect of New Cooperative Medical Scheme on health of rural elderly in 22 provinces and cities

**DOI:** 10.1186/1475-9276-13-37

**Published:** 2014-05-13

**Authors:** Ying Liang, Peiyi Lu

**Affiliations:** 1 Department of Social Work and Social Policy, School of Social and Behavioral Sciences, Nanjing University, Nanjing, Jiangsu, 210023, People’s Republic of China; 2 School of Communication and Design, Sun Yat-sen University, Guangzhou, People’s Republic of China

**Keywords:** Basic medical insurance policy, New Cooperative Medical Scheme, Health, Rural elderly, CLHLS, Longitudinal comparison, Chinese government

## Abstract

**Background:**

The alarming progression of the aging trend in China attracts much attention in the country and abroad. In 2003, the Chinese central government launched the New Cooperative Medical Scheme (NCMS) to resolve the inequity problem of health in regions with inadequate infrastructure and relative poverty. The rural elderly are the main beneficiaries of this policy; the improvement of their health through the medical insurance policy require exploration.

**Methods:**

This study used data obtained from the Chinese Longitudinal Healthy Longevity Survey (CLHLS) conducted in 2005 and 2008. Elderly people living in rural areas and aged 60 and above were screened for the investigation. A total of 8658 and 9904 elderly people were selected from 2005 and 2008, respectively. By establishing models and employing multi-logistic analysis, stereotype logistic analysis, we examined the effect of NCMS organized by Chinese government on three domains of the health of the rural elderly.

**Results:**

A total of 948 and 6361 elderly people participated in NCMS in 2005 (*n* = 8658) and 2008 (*n* = 9904), respectively. With regard to the independent variables, the number of participants in NCMS increased, whereas province distribution, gender, and years of education only slightly changed. As for the dependent variables, the rural elderly in 2005 had poor general health but good psychological health. Differences were found between different moods. Old people who engage in much outdoor activity can take care of themselves. After three-year promotion of NCMS, the differences between 2005 and 2008 indicate that the physical function of the rural elderly worsen, whereas the general health and psychological health improves.

**Conclusions:**

(1) In the 2005 data and 2008 data, result shows that NCMS participation can promote the self-rated quality and health change of the elderly. (2) After three years, the alleviation effect on anxiety and loneliness changed from insignificant to significant. Participants in NCMS have a stronger sense of uselessness, which weakens with time. (3 )NCMS participation passes the significance test in “outdoor activities” and “pick a book up from the floor” model. Elderly participants indicated higher frequencies of outdoor activities.

## Background

China is facing a serious challenge with regard to aging. A government report released in 1999 showed that China’s population over 60 had reached 127 million, which represented 10.1% of the total population [1]. In the 21st century, China’s aging trend further shows rapid growth. The first 20 to 40 years of this century are witnessing the fastest growth of the aging population. By then, the number of people aged 60 or above will have increased by 100 million every 12 or 13 years, equivalent to the total population of a populous country [2]. This growth trend and its effect should attract our attention; therefore, we investigate them in depth.

The health of the elderly worsens with age, and they typically suffer many chronic diseases [3]. Elderly patients have complicated social, nutritional, and dependency needs; thus, the burden of degenerative disease has been imposed on the agenda at all levels of government [1]. However, the Chinese medical system is not fully prepared to manage the rapid aging trend in China [4]. The health care system fails to meet the demand for health care by the aging population because of a lack of supply of medical resources [5]. The Chinese medical system must resolve many issues, including out-of-pocket payments, cost increases, and extensive inequality [6]. Important policies have been introduced to address these issues.

The relationship between the medical scheme organized by the government and the health has always been a popular topic. The health of the rural elderly, in particular, arouses great concern. The social security, policy, and welfare coverage in China rural areas are worse than those in urban areas. Health administration is limited. The health care provided to the rural elderly will be undermined if the government fails to develop an improved system of rural medical insurance. In 2003, the Chinese central government launched a policy called the New Cooperative Medical Scheme (NCMS) in rural areas. This scheme aims to safeguard the access of farmers to basic health services and alleviate the financial burden caused by sickness and poverty [7]. NCMS was introduced against the background of the Chinese market economy. It is also expected to partly resolve the inequity problem of health in regions with inadequate infrastructure and relative poverty. The effectiveness of the policy in addressing the medical insurance problem in rural areas and in improving the health of the elderly warrants investigation.

### Health conditions of Chinese elderly

Many countries are facing several problems related to aging. Among the most important of such issues is the health of the elderly. In China, the rural elderly constitute nearly 70% of the total elderly population [8]. However, the rural elderly have poor health. The rate of hospital utilization by the rural elderly is low [9], and their mental health is significantly worse than that of the urban elderly population [3]. The suicide rate of the rural elderly is three to five times that of the urban elderly as a result of illness, household conflicts, and financial difficulties [10]. The factors influencing the health of the elderly are diverse and complicated.

***First*,** the effect of family support or living arrangements on the health of the elderly has been of great concern [10-12]. Chronic disease, impaired cognitive function, and lack of mental support induce depression and anxiety, but these negative effects can be countered by family involvement [13]. Living arrangements also strongly influence the psychological health of the elderly. The importance of family in Chinese traditional culture affects the subjective well-being of the Chinese [12,14].

***Second*,** from a macro perspective, the poor health of the rural elderly is associated with the lack of a system for proper medical care in China’s urban and rural dual scheme. Huge medical expenses and a lack of insurance hinder the rural elderly from seeking medical attention because of the financial burden. Available infrastructure cannot meet the basic needs of the population and thus frustrates them [15]. Medical expenses cause Chinese rural households to become poor [16]. Socio-economic status affects the elderly’s health, of which bank savings are the most reliable predictor [17]. When the rural elderly fall ill, they are forced to forgo treatment because they cannot afford it, and thus impair their health.

***Third*,** researchers have explored the factors affecting the health of the elderly in China from different perspectives. These factors include late childbearing [18], socio-economic conditions in childhood [19], living arrangements [12,20], socio-demographic differences in terms of self-rated health [21] and mortality [22], and environmental factors [23].

These studies provide micro-individual, macro-community, and macro-environment reference perspectives on the factors that affect the health of the elderly. However, few studies have clearly defined the extent to which these factors explain the differences of their effects on health in multi-level analysis models [5]. In addition, longitudinal studies on the health of the Chinese elderly must be enriched because their health is affected not only by their living conditions but also by their previous life experiences. The health of the Chinese elderly warrants further research.

### Review of NCMS

**Figure 1:**
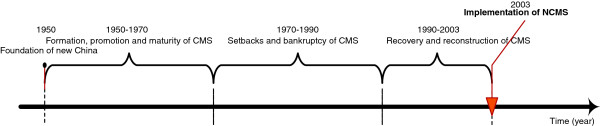
Time axis of development of CMS.

Prior to the implementation of NCMS in 2003, the cooperative medical scheme (CMS) had been established for over 60 years. CMS was established in the 1950s, when industrialization was still in its infancy, just after the foundation of new China. Figure 1 shows the development of CMS in Chinese rural areas since its establishment. Under this scheme, farmers can visit the local clinic, managed by a cooperative community in charge of production, and be charged with only minimal fees. These farmers collectively raised medical funds [24]. CMS was successful for a time, but it collapsed with the reform and opening-up in the 1980s. This collapse is attributed to two main factors: the lack of voluntary community financing and ineffective government policies [25]. After the bankruptcy, the health care system became unable to protect Chinese farmers. In 1998, only 9.5% of farmers were insured.

The Chinese government has rehabilitated and reconstructed the system of rural medical security since the 1990s. After years of piloting in multiple areas, the central government gradually developed the NCMS. Although this scheme is organized by the local government, rural residents participate voluntarily. This medical scheme supported by mutual aid focuses on planning for serious diseases and is financed by individuals, collectives, and the government. Table 1 compares the various highlights of CMS and NCMS policies. The innovation of NCMS lies in its aspects of serious coordination and mutual aid. This scheme also represents the commitment of the government to bear the main financial responsibility for medical support for the first time, through government revenue [26].

**Table 1:** Comparison between CMS and NCMS organized by Chinese government

	**CMS**	**NCMS**
Source official document	Prospectus (draft) of cooperative medical scheme for rural areas [27]	The State Council forwards the notice released by the Ministry of Health regarding the establishment of the New Cooperative Scheme for rural areas.
Year of issue	December 15, 1979	January 16, 2003
Goal	To protect the health of community members and develop agricultural production	To relieve the financial burden on farmers because of illness and improve their health
Principle		Voluntary participation, multi-party financing, and fixed income support. A pilot is conducted before the scheme is established gradually
Organization and management	The production team constructs cooperative medical stations (clinics) and establishes a committee composed of cadres, representatives, and health staff.	The county (district) generally initiates coordination while the province (city) sets up a coordination group.
Financing	Raised by individuals and organizations (Community chest) that participate in CMS	Raised through individual contributions, collective support, and government funding
Management of funds	Managed by the production team or community health care workers	Managed by the NCMS management committee and its agencies

***First*,** the controversial effect of NCMS has been the focus of many studies since the implementation of the scheme. On the one hand, the coverage rate of Chinese health insurance significantly increased from 28% in 2004 to 49% in 2006 [28]. A Chinese scholar assessed the implementation effect of the policy in 2007 and found that the policy had been successful despite the presence of several problems [24]. On the other hand, other researchers believe that NCMS has had only marginal effects and still fails to lessen the burden imposed by the health care system, which requires out-of-pocket expenses from many rural households [29-32]. The average reimbursement percentage of NCMS is 17.8%, which indicates merely modest economic protection [29]. Reimbursement is also limited for rural–urban migrants who avail of hospital services [33].

***Second*,** several studies have examined the health-seeking behavior of farmers under the NCMS and have found that age and relative income are negative influential factors. For example, the average reimbursement rate and daily family expenses are strongly associated with hospital choice [34]. Among low-income groups, the price elasticity of demand for outpatient service is high [35], but the utilization rate of outpatient service, medical expenses, and catastrophic expenditure only slightly change under NCMS [36,37]. However, NCMS has increased the utilization rate of inpatient service [36] and has improved preventive care, especially in terms of general physical examination [32]. This scheme protects poor inpatients with respect to health care expenses but has accomplished its goal only partially [38]. These studies have explored economic factors in the development of NCMS, providing excellent references on promoting the implementation effect of NCMS and on improving the quality of medical care in rural areas.

***Third*,** Chinese scholars have discussed the scheme design, implementation problems, and measures to reform and improve NCMS. However, performance evaluation methods still require dynamic comparative research [39]. Over 10 years has passed since the implementation of NCMS, and a large amount of empirical data are required to expand and enrich research on the effectiveness and influence of the scheme. The policy primarily aims to protect and enhance the health of Chinese farmers, but whether and how NCMS affects the health of insured farmers have not been dynamically studied in detail.

### Research questions and objectives

Researchers have noted the influence of medical care schemes on health. The traditional system of medical care seriously affects the (poor) health of the elderly [5]. Since 2003, NCMS has been introduced to the vast rural areas in China to benefit the nearly 10 million rural populations. This study considers the following questions: Can the NCMS achieve its original intention, especially with respect to the rural elderly as the target subjects? Does it help to alleviate the inequity problem? What is the mechanism of the effect of NCMS on health?

This research focuses on the influence of NCMS organized by Chinese government on the health of the rural elderly. By using an open database, the study establishes models and employs regression analysis to explore the effect mechanism. It aims to determine how NCMS affects each domain of elderly health. Thus, the research objectives are (1) to explore how a large-scale policy on medical care influences the health of its target objects, (2) to discuss the extent of the effect of a new medical care policy in different periods and understand its effect mechanism on the health of the rural elderly, and (3) to advise policy decision makers and medical staff regarding health domains that can be enhanced in the NCMS.

## Methods

### Data resources

The study used data obtained from the Chinese Longitudinal Healthy Longevity Survey (CLHLS) in 2005 and 2008. CLHLS is a large-scale multidisciplinary, longitudinal follow-up survey of the elderly. The baseline survey started in 1998, covering individuals aged 65 and above in 801 counties of 22 Chinese provinces and cities. The current study aims to explore the influential factors in the health of the Chinese elderly, including demographic characteristics, cognitive ability, and lifestyle, to strengthen the scientific research and policy analysis of healthy aging [40,41].

The study mainly surveyed households, and the follow-up survey was divided into two categories based on respondent survival: For surviving respondents, investigators visited their homes and asked them to fill out the questionnaire; for the respondents who died, investigators also visited their homes and asked their families to fill out the designated form to collect information on these respondents before they died.

Follow-up surveys of CLHLS were conducted in 2000, 2002, 2005, 2008–2009, and 2011–2012. NCMS was not launched in Chinese rural areas until 2003; therefore, this study obtained samples from 2005 and 2008 for comparative analysis. The total sample sizes from the 2005 and 2008 data were 15,638 and 16,540, respectively.

Given the special nature of the research object, we screen out people aged 60 and above who lived in rural areas during the investigation. Finally, 8658 and 9904 cases were selected from the 2005 and 2008 data, respectively. The overall quality of the data collected by CLHLS is more satisfactory than that of data collected by other major international surveys on aging [42].

### Research hypotheses

For the Chinese central government, NCMS is essential to resolve the problematic medical care system in rural areas. This policy aims to benefit farmers, who dominate the total population. This research thus explores the effect of NCMS on all domains of elderly health and its operation mechanism.

The aim of the policy to improve the social capital of rural areas is promising with respect to improving health and well-being [43]. A survey conducted in Shandong Province, China, in 2011 showed that 82% of the population were willing to continue participating in NCMS and that most of them were optimistic regarding the policy [44]. Based on the policy objectives and the attitudes of the target people, NCMS organized by the government may positively affect the overall health perception of the elderly. Thus, we propose the following hypotheses:

#### Hypothesis 1

NCMS significantly affects the general health of the elderly.

#### Hypothesis 2

NCMS significantly affects the psychological health of the elderly.

To determine elderly health, we strictly observe physical function. The rural elderly are more active than the urban elderly in daily life; however, the daily activities and physical performance of the rural elderly decline significantly with age [45]. Functional ability is negatively associated with the quality of life of the elderly. Those with chronic disease rated their health as poor [46]. An important effect of NCMS is reflected in the increased utilization of medical services by the participants. Therefore, improving the utilization of medical services is an important channel through which NCMS can affect the health of participants [37]. When the rural elderly fall ill, NCMS organized by the government should support them financially so that they receive proper medical aid for recovery. We thus propose the following hypotheses:

#### Hypothesis 3

NCMS significantly affects the physical functions of the elderly.

### Variables

#### Independent variable

The independent variables were classified into three categories: NCMS participation, basic information, and medical service utilization.

NCMS participation was the key independent variable in this study. To distinguish the effect of NCMS from those of other medical insurance, we recoded cases that did not possess any medical insurance as 0 and recoded those that participated only in NCMS as 1. The numbers of cases that participated only in NCMS were 948 and 6361 in 2005 and 2008, respectively. Basic information included age, gender, years of education, and province of residence during the investigation. Medical care utilization consisted of two variables: the current availability of adequate medical services and medical expenses in the previous year. The 2005 and 2008 data are described in detail and compared in Table 2.

**Table 2:** Description and comparison of independent variables

		**NCMS participation**	**Province**	**Age**	**Gender**	**Years of education**	**Current utilization of adequate medical services**	**Medical expenses in previous year**
Mean	2005	0.117	1.95	86.68	1.58	1.39	1.15	810.59
	2008	0.71	1.91	87.03	1.58	1.51	1.09	1082.98
Plural	2005	0	1	100	2	0	1	0
	2008	1	1	100	2	0	1	0
Minimum	2005	0	1	63	1	0	1	0
	2008	0	1	60	1	0	1	0
Maximum	2005	1	3	117	2	25	2	100000
	2008	1	3	116	2	25	2	100000
Skewness	2005	2.389	0.096	−0.122	−0.321	3.342	1.983	14.025
	2008	−0.904	0.168	−0.292	−0.33	2.178	2.765	14.444
Standard error of skewness	2005	0.027	0.026	0.026	0.026	0.026	0.026	0.027
	2008	0.026	0.025	0.025	0.025	0.025	0.025	0.025
Kurtosis	2005	3.709	−1.617	−1.059	−1.897	6.601	1.934	379.101
	2008	−1.183	−1.498	−0.885	−1.891	5.385	5.644	316.842
Standard error of kurtosis	2005	0.054	0.053	0.053	0.053	0.053	0.053	0.053
	2008	0.052	0.049	0.049	0.049	0.049	0.049	0.050

### 2005 data

The mean value of NCMS participation was 0.117, and the plural was 0. In the initial stage of NCMS, each province, autonomous region, and municipality must select at least two to three counties (cities) as pilots to gain experience before extending the implementation. Therefore, only a small number of rural elderly participated in NCMS in 2005. The provinces were divided into three dummy variables according to location: east = 1, central = 2, and west = 3. As observed from the mean value and plural of the 2005 data, most of the elderly lived in the eastern and central areas.

The mean value of age was 86.68, and the gap was between 63 and 117. Participants aged under 69 accounted for 10.9% of the total sample; those aged 70 to 79 and 80 to 89 constituted 21.3% and 24.5%, respectively. Those aged over 90 dominated the sample at 43.3%. The skewness and kurtosis of age variables were both negative, thus showing that age deviated negatively. The skewness was also not as steep as the normal distribution. More women (57.9%) participated in NCMS than men (42.1%), consistent with the distribution of the total population. The mean value of years of education was 1.39, indicating that educational level is low.

As for the item “current utilization of adequate medical services”, 1 and 2 represented yes and no. Of the respondents, 85.2% felt that they enjoy adequate medical care, and only 14.8% complained about their utilization of medical services. As illustrated in Table 2, the medical services enjoyed by the elderly were reasonable and positive. The gap between the maximum and minimum values of the variable “medical expenses in the previous year” peaked at 100,000 RMB. The kurtosis confirmed that the distribution of medical spending was rather steep, showing great differences in medical expenses among the cases. This result indicated the extraordinarily high cost of health care in China, and the onset of serious illness can result in the poverty of peasant families with modest economy.

### 2008 data

The mean value of the variable “NCMS participation” was 0.71, and the plural was 1, revealing that the number of NCMS participants increased greatly over time. The distribution of provinces in 2008 only slightly differed from that in 2005. Although the mean value dropped to 1.91, most of the respondents were distributed in the eastern areas.

The mean value of age in 2008 was 0.35 greater than that in 2005, matching the characteristics of the sample. Gender distribution in 2008 was also consistent with that in 2005. Years of education in 2008 were higher than that in 2005; however, the educational level of the rural elderly remained low. The skewness of years of education was 2.178 (>0), whereas its kurtosis was 5.383 (>1), indicating that age deviated positively. The skewness was steeper than the normal distribution.

The mean value of the variable “current utilization of adequate medical services” in 2008 was lower than that in 2005. However, additional evidence is required to relate this occurrence to NCMS implementation. “Medical expenses in the previous year” in 2008 differed from that in 2005.

As per the comparison of the 2005 and 2008 data, the number of participants in NCMS increased. Province distribution, age, and years of education remained constant.

**Figure 2:**
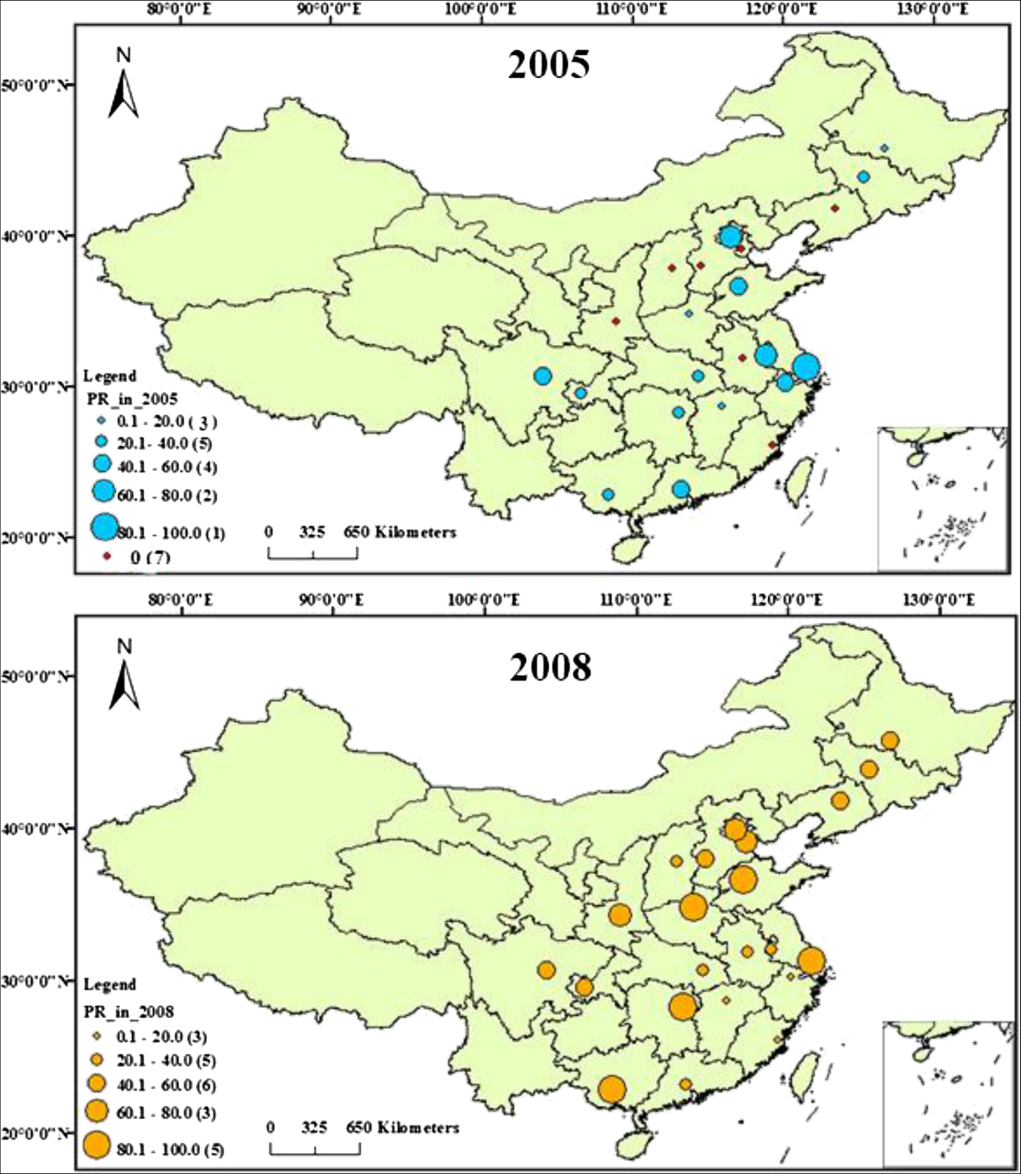
Comparison of NCMS participation rate in sampled provinces in CLHLS database.

Figure 2 shows the NCMS participation rate of the rural elderly in each sampled province of China in the CLHLS database from 2005 (above) to 2008 (below). The red point indicates that the participation rate is 0%. The area of a circle is positively related with the range size of participation rate. The smallest circle indicates that the range of participation rate is 0.1% to 20.0%. The largest circle indicates that the range of participation rate is 80.1% to 100%. Comparison shows that seven provinces have 0% participation rate in the 2005 data, whereas this did not occur in the 2008 data, which indicates that provincial participation in NCMS was encouraged during the three-year gap. In addition, the above figure has more circles with larger areas, indicating that in general, the participation rate of every sampled province in China had been improved. That is, NCMS organized by the Chinese government was accepted well.

### Dependent variables

The dependent variable was the health of the rural elderly. The 36-item short-form health survey scale (SF-36) divided the measures of health-related quality of life (HRQOL) into eight domains, namely, physical function, physical role, bodily pain, general health, vitality, social function, emotional role, and mental health [47,48]. This study referred to this scale and measures the health of the elderly in three domains: general health, psychological health, physical function. Table 3 describes these domains in detail.

**Table 3:** Dependent variables selected from CLHLS

**General health**	**Psychological health**	**Physical function**
B11: Self-rated quality of life	B23: Feel fearful or anxious	D11b: Do you do any outdoor activities at present
B12: Self-rated health	B24: Feel lonely and isolated	G9: Are you able to stand up from sitting in a chair?
B121: Do you feel any change of your health since last year	B26: Feel useless with age	G11: Are you able to pick a book up from the floor?

The domain of general health was made up of three types of variables: self-rated quality of life (b11), self-rated health (b12), and “do you feel any change of your health since last year?” (b121). The items related to self-rated quality of life and self-rated health can be rated as very good, good, fair, poor, very poor, and cannot answer according to the feelings of the respondents. Items under “cannot answer” were classified as missing data in data preprocessing. There are five options in the item health change (b121), they are “much better”, “slightly better”, “almost the same”, “slightly worse”, “much worse”.

Psychological health was divided into three kinds of variables: feel fearful or anxious (b23), feel lonely and isolated (b24), and feel useless with age (b26). Each item has five options: “always”, “often”, “sometimes”, “seldom” and “never”.

Physical function is also composed of three variables: “do you do any outdoor activities at present?” (d11b), “are you able to stand up from sitting in a chair?” (g9), and “are you able to pick a book up off the floor?” (g11). Whether the elderly engage in any outdoor activities (d11b) can be judged from the frequency illustrated by five options: “almost everyday”, “not every day, but at least once a week”, “not every week, but at least once a month”, “not every month, but sometimes” and “never”. Their ability to stand up from sitting in a chair (g9) has three options: “yes, without using hands”, “yes, using hands” and “no”. Their ability to pick up a book from the floor (g11) has three options: “yes, standing”, “yes, sitting” and “no”.

Descriptive statistical analysis and a comparison of the variables in 2005 and 2008 are listed in Table 4.

**Table 4:** Descriptive statistical analysis and comparison of variables in 2005 and 2008

		**B11**	**B12**	**B121**	**B23**	**B24**	**B26**	**D11b**	**G9**	**G11**
Mean value	2005	2.40	2.60	3.35	3.99	3.87	2.44	2.93	1.42	1.47
	2008	2.40	2.58	3.32	3.97	3.87	3.11	3.03	1.46	1.50
Plural	2005	2	2	3	4	5	1	1	1	1
	2008	2	2	3	5	5	3	5	1	1
Minimum	2005	1	1	1	1	1	1	1	1	1
	2008	1	1	1	1	1	1	1	1	1
Maximum	2005	5	5	5	5	5	5	5	3	3
	2008	5	5	5	5	5	5	5	3	3
Skewness	2005	0.404	0.260	−0.136	−0.614	−0.666	0.364	0.096	1.253	1.160
	2008	0.370	0.204	−0.126	−0.714	−0.608	0.037	-0.076	1.136	1.056
Standard error of skewness	2005	0.027	0.027	0.028	0.028	0.028	0.028	0.026	0.026	0.026
	2008	0.026	0.026	0.027	0.027	0.027	0.027	0.025	0.025	0.025
Kurtosis	2005	0.211	−0.405	0.433	−0.100	−0.107	−0.900	−1.804	0.360	0.001
	2008	0.200	−0.491	0.456	−0.067	−0.364	−0.792	−1.842	0.082	0.082
Standard error of kurtosis	2005	0.055	0.055	0.055	0.056	0.056	0.056	0.053	0.053	0.053
	2008	0.053	0.053	0.053	0.054	0.054	0.054	0.049	0.049	−0.249

Note: b11: Self-rated quality of life;b12: Self-rated health; b121: do you feel any change of your health since last year; b23: Feel fearful or anxious; b24: Feel lonely and isolated; b26: Feel useless with age; d11b: Do you do any outdoor activities at present?; g9: Able to stand up from sitting in a chair?; g11: Able to pick up a book from the floor?

### 2005 data

For the variable b11, mean value = 2.40, plural = 2, minimum value = 1, maximum value = 5, skewness = 0.404, and kurtosis = 0.211, thus indicating that some of the respondents felt that they were healthy, whereas some others considered themselves to be unhealthy. Nevertheless, most of the respondents rated their health as good. The results of self-rated health and quality of life were identical in terms of the plural, maximum, and minimum values. However, the mean value increased to 2.60, showing that the self-rated health of the rural elderly was slightly poorer than their self-rated quality of life. The mean variable b121 was 3, which illustrates that most respondents did not experience changes in their health. In general, the health of the rural elderly in 2005 was poor.

Variables b23, b24, and b26 were used to investigate psychological health. High value indicated the excellent psychological health of the respondent. As observed in Table 4, the mean values of feelings of fear, anxiety, and loneliness were high, whereas that of sense of uselessness is low. This result reveals that the most serious negative emotion is sense of uselessness. The plural confirmed this finding. Overall, the psychological health of the rural elderly in 2005 was good. Differences were observed between the three kinds of emotions.

As for the physical function, apart from variable d11b, the kurtoses of variables g9 and g11 were above 0, suggesting that the distribution of the variable d11b is gentler than the normal distribution. This result indicates that the rural elderly can engage in outdoor activities and take care of themselves.

### 2008 data

Overall, the results of dependent variables in 2008 only slightly differed from those in 2005. The skewness values of b121, b23, and b24 were negative, showing that these three variables were negatively skewed in terms of similarity. The kurtosis values of variables b12, b23, b24, b26, and d11b were negative, whereas those of other variables were positive, indicating that the distributions of variables b12, b23, b24, b26, and d11b were flat. Compared with the 2005 data, the 2008 data show that in the general health domain, the mean values of b12 and b121 decline, indicating that the general health of the elderly improved; in the psychological health domain, the plural of b23, b26 and the mean value of b26 increase, indicating that the psychological health of the elderly promoted; in the physical health domain, the mean values of three variables increase, indicating that the physical function declined.

### Analysis methods

There are three dependent variables. All of them are categorical variables and their answers include the orders of good and bad. Based on the features of dependent variables, we decided to use a stereotype logistic model. The model has two features: (1) the orderliness of dependent variables, that is, the options of dependent variables have certain orders in different categories based on classification. For example, the categories of self-rated health are categorized as “very good”, “good”, “fair”, “poor” and “very poor”. The significance test of multivariate differences should be conducted following each categorical variable [49]; (2) the model assume that the curve slope of categorical regression should be the same and also allows the coefficients of variables to indicate differences in different categories [50-52]. This model deepens the discussion of certain details in the proportional odds and multinomial logit model. It retains the general interpretation of proportional odds and multinomial logit model and is more flexible and convenient [53]. The formula of stereotype logistic is as follows:

$$\mathrm{Pr}\left(Y=y_{s}|x\right)=\frac{\exp\;\left(\alpha_{s}+\varphi_{S}\beta^{'}x\right)}{\sum_{l=1}^{k}\exp\;\left(\alpha_{l}+\varphi_{l}\beta^{'}x\right)},\;s=1,\;\ldots \;,\;k$$

Among these, β is the coefficient to be estimated of independent variable x. “k” is the intersection of dependent variables. α_s_, α_l_ comprise the intersection of model [50]. In the regression, we consider the last or first option of independent variables as the conference variable. Apart from the interval variables, other independent variables are set as the dummy variables. The reference variables are set as 0. Thus, k-1 odd ratio (OR) values can be obtained from any explanatory variables, indicating the changes of OR in each level. In addition, the impact direction of independent variables on dependent variables can be estimated from the positive or negative symbols of OR value [49].

Considering that the dependent variables of stereotype logistic regression are ordinal variables, they could be considered extensions of multinomial logistic regression [54]. However, it has fewer parameters compared with multinomial logistic regression. Considering that we could not obtain the majority of OR values of scale factors hidden by automatic calculation, fewer parameters do not result in the decline of interpretation difficulty [55]. This study calculates the hidden OR value via e^exp^. We can use Pearson of Hosmer-Lemeshow or the poisson goodness of fit to test the regression results [56].

Psychological health has three dependent variables. All of the dependent variables are categorical variables and include positive and negative orders. Thus, the stereotype logistic regression model is used.

Physical health also has three dependent variables. The dependent variables “d11b” used stereotype logistic regression model; and the variable “g9” used multinomial logistic regression model. In the multinomial logistic model, no requirements are needed for the rank orders of dependent variables. In each model, the measurement software takes the last category of dependent variables as the reference category by default. All its coefficients are 0. Therefore, when k indicates the number of independent variables, j indicates the number of the dependent variable categories, and the number of parameters to be estimated is k (j-1).

## Results

### General health

The stereotype logistic regression results of general health domain can be seen from Table 5. Its relationships with NCMS participation, utilization rate of medical services and basic information are as follow:

a) NCMS Participation

In the 2005 data, NCMS participation generally does not have significant impact on general health. Only the self-rated quality of life passes the significance test. The value of OR is 0.324, indicating that the probability of the self-rated quality of life of the elderly participants decreasing one level is 0.324 times that of non-participants. This indicates that NCMS participation can promote the self-rated quality of life of the elderly. In the 2008 data, the OR value of participation in NCMS to the self-rated quality of life of the rural elderly is 0.671, indicating that the probability of the self-rated quality of life of elderly participants decreasing one level is 0.671 that of non-participants. This indicates that NCMS participation can promote the improvement of the general health of rural elderly.

b) Utilization rate of medical services

The variables of “Medical expenses in previous year” and “Utilization of adequate medical services” are used to measure the utilization rate of medical services. As illustrated by the results, these two independent variables pass the significance test in all six models. Among them, the OR values of “Utilization of adequate medical services” are below 1, indicating that the general health of the elderly who obtain adequate medical services is better than those who do not obtain these services.

c) Basic information

Generally speaking, the general health of the elderly living in eastern China is better than that of those living in Western China (OR < 1), and both of them pass the significance test. Notably, when compared with the 2005 data, the 2008 data has smaller OR value, indicating that the differences between eastern and western areas are becoming more obvious with the promotion of NCMS. In addition, the OR value between middle and western areas shows instability, indicating that rural areas in Middle and Western China should strengthen the promotion of NCMS.

In addition, the years of education pass the significance tests when b11 and b12 are dependent variables. Results show that scores of self-rated quality of life and self-rated health of the rural elderly are higher with education. This may be because that the elderly with higher education are characterized by better physical fitness and a more positive attitude towards life.

**Table 5:** Stereotype logistic regression of the general health

	**Model 1**		**Model 2**		**Model 3**	
	**b11**		**b12**		**b121**	
	**2005**	**2008**	**2005**	**2008**	**2005**	**2008**
	**Coefficients**	**Coefficients**	**Coefficients**	**Coefficients**	**Coefficients**	**Coefficients**
NCMS participation (no = 0)	−1.125***	−0.399**	−0.300	−0.158	0.047	−0.376***
Medical expenses in previous year	0.000**	0.000*	0.000***	0.000***	0.000***	0.000***
Utilization of adequate medical services (no = 0)	−3.810***	−4.358***	−2.306***	−2.194***	−1.305***	−1.514***
Province = eastern (western = 0)	−0.545**	−1.453***	−0.369**	−1.728***	0.468***	−0.987***
Province = middle (western = 0)	−0.576***	−0.186	0.168	−0.853***	0.523***	−0.456***
Male (female = 0)	0.285*	0.388**	−0.071	−0.165	0.042	−0.212*
Age	−0.012*	−0.011	0.010*	0.006	0.023***	0.015***
Years of education	−0.091***	−0.112***	−0.083***	−0.049*	−0.027	−0.015
Df	8	8	8	8	8	8
Wald chi(2)	215.74	239.52	209.67	328.31	32.77	39.67

Note: ***indicates p < 0. 001; **p < 0.01; *p < 0.05. b11: Self-rated quality of life; b12: Self-rated health; b121: Do you feel any change of your health since last year.

### Psychological health

The stereotype logistic regression results of psychological health domain are shown in Table 6. The details of three variables are as follow:

**Table 6:** Stereotype logistic regression of psychological health

	**Model 4**		**Model 5**		**Model 6**	
	**b23**		**b24**		**b26**	
	**2005**	**2008**	**2005**	**2008**	**2005**	**2008**
	**Coefficients**	**Coefficients**	**Coefficients**	**Coefficients**	**Coefficients**	**Coefficients**
NCMS participation (no = 0)	0.181	0.647***	0.209	0.423***	−0.348**	−0.411***
Medical expenses in previous year	−0.000*	−0.000***	−0.000	−0.000	0.000	−0.000*
Utilization of adequate medical services (no = 0)	0.845***	1.534***	1.536***	2.068***	−0.336**	1.486***
Province = eastern (western = 0)	−0.340**	0.700***	−0.357**	0.718***	−1.001***	−0.541***
Province = middle (western = 0)	0.384**	−0.480***	0.072	−0.364***	−0.900***	−1.484***
Male (female = 0)	0.480***	0.950***	0.450***	0.362	−0.572***	0.303***
Age	−0.006	−0.023***	−0.029***	−0.051***	0.058***	−0.039***
Years of education	0.056**	−0.013	0.070***	0.012	−0.105***	0.079***
Df	8	8	8	8	8	8
Wald chi(2)	19.00	155.41	126.91	233.54	290.31	335.98

Note: ***indicates p < 0. 001; **p < 0.01; *p < 0.05. b23: Feel fearful or anxious;b24: Feel lonely and isolated;b26: Feel useless with age.

#### Fear and anxiety

In the 2005 data, NCMS participation does not significantly affect fear and anxiety.

In the 2008 data, NCMS participation significantly affects fear and anxiety. The OR value is 1.910, indicating that the probability of fear and anxiety decreasing in elderly participants is 1.910 times that of non-participants. Thus, NCMS can alleviate fear and anxiety.

The results indicate that the alleviation effect of NCMS on fear and anxiety changed from insignificant to significant. NCMS organized by Chinese government is an important part of the social security system, and its implementation provides additional protection to the rural elderly. In times of sickness, they obtain increased financial security, which enables them to receive proper medical care. As a result, they feel less fear and anxiety.

#### Loneliness

In the 2005 data, loneliness does not pass the significance test whereas it passes in the 2008 data. The results indicate that NCMS positively alleviates symptoms of loneliness. After three years of promotion, the alleviation effect of NCMS on the symptoms of loneliness is greater and passes the significance test.

#### Sense of uselessness

The sense of uselessness passes the significance test in both the 2005 and 2008 data. The OR value of 2005 data is 0.706 whereas that of 2008 data is 0.663. In 2005, the probability of the sense of uselessness decreasing in elderly participants is 0.706 times that of non-participants. In 2008, the probability of the sense of uselessness decreasing in participants is 0.663 times that of non-participants. The results indicate that elderly participants have a stronger sense of uselessness. However, negative impact weakens with time.

### Physical function

The stereotype logistic and multinomial logistic regression results of physical function domain are illustrated in Table 7. Its relationships with NCMS participation and basic information are as follow:

(1) NCMS participation

NCMS participation passes the significance test when the dependent variables are “d11b and g11”. Elderly participants indicated higher frequencies of outdoor activities than non-participants. This result indicates that the awareness of elderly participants is stronger than that of non-participants. Therefore, the elderly should be taught to raise the awareness of health’s importance when NCMS is implemented.

(2) Gender

Gender passes the significance test. In the model in which variables g9 and g11 are dependent variables, the coefficients are positive and the OR value is above 1. This indicates that males have better physical functions than females do.

(3) Age

Age passes the significance test in all six models. In the model in which variable d11b is the dependent variable, the coefficient is positive, indicating that outdoor activities decrease with age (in the questionnaire, greater options lead to a smaller frequency of outdoor activities.). The result may be related to physiological laws. The physical function of the elderly declines with age. The frequency of outdoor activities is also reduced because of pain or illness.

(4) Years of education

Years of education are also proportional to the frequency of outdoor activities, and are associated with the exercise awareness of the elderly. The elderly with more years of education may be more aware of the importance of outdoor activities.

**Table 7:** Stereotype logistic and multinomial logistic regression of physical function

	**Model 7**		**Model 8**		**Model 9**	
	**Stereotype logistic**		**Multinomial logistic**		**Multinomial logistic**	
	**d11b**		**g9**		**g11**	
	**2005**	**2008**	**2005**	**2008**	**2005**	**2008**
	**Coefficients**	**Coefficients**	**Coefficients**	**Coefficients**	**Coefficients**	**Coefficients**
NCMS participation (no = 0)	−0.331***	−0.458***	0.219	0.083	0.368**	0.163*
Medical expenses in previous year	0.000***	0.000***	0.000***	0.000***	0.000***	0.000***
Utilization of adequate medical services (no = 0)	−0.338***	−0.118	0.870***	0.620***	0.760***	0.637***
Province = eastern (western = 0)	0.129	0.494***	−0.119	−0.611***	−0.610***	−0.686***
Province = middle (western = 0)	0.578***	−0.431***	0.017	−0.242*	−0.317**	−0.117
Male (female = 0)	−0.282***	0.316***	0.374***	0.442***	0.565***	0.524***
Age	0.052***	0.047***	−0.091***	−0.109***	−0.119***	−0.137***
Years of education	−0.052***	−0.027*	0.006	0.006	0.024	0.026
NCMS participation (no = 0)			−0.071	0.197*	−0.054	0.354***
Medical expenses in previous year			0.000***	0.000***	0.000***	0.000***
Utilization of adequate medical services (no = 0)			0.386***	0.481***	0.376***	0.411***
Province = eastern (western = 0)			0.313**	−0.302**	−0.610***	−0.687***
Province = middle (western = 0)			0.590***	0.157	−0.103	0.082
Male (female = 0)			0.019	0.182	0.168	0.212*
Age			−0.006	−0.015**	−0.034***	−0.042***
Years of education			−0.008	−0.009	0.002	−0.029

Note: ***indicates p < 0. 001; **p < 0.01; *p < 0.05. d11b: Do you do any outdoors activities at present?; g9: Able to stand up from sitting in a chair?; g11: Able to pick up a book from the floor?

## Discussion

The aging trend in China is progressing at an alarming rate, and this phenomenon has attracted much attention in the country and abroad. The health of the elderly is an important issue; thus, the Chinese government launched the NCMS, which aims to insure the health of the population in the rural areas of the country and further to resolve the inequity problem of health in regions with inadequate infrastructure and relative poverty, in 2003. Given that the rural elderly are the target beneficiaries, the guarantee and promotion of their health are worthy of in-depth discussion. This study obtains data from CLHLS in 2005 and 2008 to determine the effect of NCMS on the health domains of the rural elderly and its effect mechanism. This research aims to confirm whether NCMS, which is a basic policy of medical insurance that extensively covers the most populous regions of China, can improve the health of its target objects or help to alleviate the inequity problem? By establishing models and using regression analysis, this study draws the following conclusions:

1. The number of NCMS participants increased between 2005 and 2008. In the three years following the implementation of NCMS, the coverage of medical care service in rural areas rose. Province distribution, gender, and years of education remained constant.

Stereotype logistic regression of the general health domain indicates that NCMS participation can improve the general health of rural elderly. The general health condition of the elderly with adequate medical services is better than those without. Furthermore, general health indicates significance in these areas as well as education. Therefore, Hypothesis 1 is supported.

Since the implementation of NCMS, the coverage rates of medical insurance in rural and urban areas have become similar and benefited millions of rural Chinese [28]. The Rural Mutual Health Care scheme, another rural medical insurance scheme with less coverage than NCMS, positively influences the health of its participants [57]. The positive effects of the policy are understandably embodied by its capability to enhance the general health of the rural elderly. The positive effect of utilization of adequate medical services is consistent with common sense, that is, it can effectively promote the health of vulnerable or marginalized groups [58].

2. After three years of promotion, the alleviation effect on anxiety changed from insignificant to significant. The relief effect of NCMS on loneliness symptoms is greater. Elderly participants in NCMS have a stronger sense of uselessness, which weakens with time. The analysis shows that the effect of NCMS on psychological health may vary with the category. NCMS organized by Chinese government can effectively ease fear, anxiety, and loneliness in the rural elderly; however, it promotes sense of uselessness. Thus, the effect of NCMS on the psychological health of the rural elderly is not always positive or optimistic. Thus, Hypothesis 2 is supported.

The results show that NCMS is statistically significant in mitigating the loneliness of the rural elderly. Depression symptoms are common among the Chinese rural elderly and are strongly associated with living alone and low functional recognition [59] and quality of life [60]. In this research, we examine loneliness as another variable of psychological health. The findings reveal that NCMS can ease the loneliness of the rural elderly and thus improve their quality of life [61].

It also may be related to the lifelong living conditions of the Chinese rural elderly; scholars have described work conditions as ceaseless toil. Failing health does not hinder continuous hard work [62]; thus, the elderly fail to enjoy their twilight years as a result of labor. Many old people must depend on personal earnings in old age and work until they are no longer capable [63]. Thus, in times of illness, the rural elderly are not only required to spend much for medical treatment but must also seek relief from others (such as their offspring or local government). This dependence conflicts with their former self-sufficiency and results in a sense of uselessness. Therefore, they are prone to thinking that they are a burden to others.

3. Elderly participants in NCMS have stronger awareness of outdoor activities than non-participants do. Gender, age, and education positively affect physical function, and all of them pass the significance test. Men have better physical functions than women do. The frequency of outdoor activities decreases with age. Furthermore, the frequency of outdoor activities is proportional to the education. Therefore, Hypothesis 3 is supported.

In terms of NCMS promoting awareness of outdoor activities with the elderly, the program has partially achieved its goal to improve the physical function of the elderly. Previous studies have found that the implementation of a medical insurance scheme positively influences the mobility and usual activity of people aged 55 and above [57]. Certain programs aimed at improving health self-management can remarkably promote the health of beneficiaries [64] because truly improving health requires a decreased reliance on medical services [65]. We assume that when NCMS promotes policy, it has publicized the importance of health to the rural elderly, therefore raising their awareness. Regardless of the reimbursement or compensation granted to ill people, maintaining a healthy body and reducing the utilization of medical care remains the optimal solution. Participation in outdoor activities is a direct and effective method.

Overall, the results of this study are optimistic. NCMS organized by Chinese government promotes the health of the rural elderly, and insurance coverage in rural areas has increased over time. The policy is effective in terms of its targets. More important, NCMS enhances general health and physical function. The policy can affect psychological health through its influence on physical health [48] and can affect the performance of daily activities among vulnerable groups [66]. These findings suggest that NCMS positively influences controllable health indicators. Therefore, this development direction should be maintained in the future.

Meanwhile, the conclusions of this study show that NCMS organized by Chinese government does not always promote all domains of the psychological health of the rural elderly—which is an undesirable outcome. Although daily activity is associated with the quality of life of the elderly [60], cognitive function is also an important factor [11]. Low cognitive function is associated with limited social support, and this relationship affects the psychological health of the elderly [67]. Good self-rated health, which denotes personal attitude toward health, is related to robust psychological health [68]. Despite the importance of psychological health on the overall perception of health, NCMS does not significantly promote it. NCMS can therefore improve in this sense, and sensitive and effective measures should be considered.

Practical research data indicate that outdoor activities or leisure activities have positively affect the health of the elderly [69,70]. Leisure activities can reduce the risk of dementia of the elderly [71-73]. Many elderly are afraid to go out because of their fear of falling, which may actually increase the self-reported risk of walking [74]. In fact, outdoor activities can strengthen the control of emotional actions, hence improving the subjective well-being [75]. Outdoor activities positively affect the psychological health of the elderly because these involve social interaction. Outdoor activities also strengthen self-worth and promote the physical functions [76]. Therefore, the publicity and promotion of NCMS can indirectly improve the rural elderly’s awareness of outdoor activities, indicating positive impact on their health.

Therefore, we can conclude that to a certain extent, NCMS organized by Chinese government helps to alleviate the inequity health problem of the elderly in Chinese rural areas although this effect is not obvious. Based on the analysis of how NCMS affects the health of the rural elderly, we present some suggestions to decision makers or practitioners. ***First,*** the promotion effects of NCMS on general health and physical health can be effectively consolidated to improve the system of primary health care. Among the elderly, chronic diseases are prevalent, and the costs of medical care are high. Therefore, they require long-term medical treatment, prevention, and care. Focusing on medical care in the community is a good direction for future development [77] and allows the elderly to recuperate and receive comprehensive care in familiar environments. ***Second,*** psychological factors should be properly considered in medical assistance and support for the rural elderly [78]. The alleviation of the costs of health care for the rural elderly should not be limited to the NCMS; the pension system in rural areas should effectively coordinate with and complement this policy. For instance, the establishment of a pension system that provides minimum living security to the rural elderly as in the pension system in cities may ease the sense of uselessness. ***Third,*** knowledge propaganda should be strengthened and maintained. Reasonable understanding of participants regarding the use of health resources should be enriched. The importance of outdoor activities, as a side effect of NCMS publicity, should be given additional emphasis. By strengthening elderly awareness on the importance of prevention via exercise as opposed to reliance on medical care after falling sick, the effect of NCMS can be maximized.

This study has several limitations. ***First,*** given the limitations of the questionnaire designed by open databases, the variables were selected based on the HRQOL scale; however, the health of the elderly can be measured on a mature scale, such as the SF-36, SF-12 and short version of World Health Organization Quality of Life questionnaire (WHOQOL-BREF) scales, etc. [79-81]. ***Second,*** this research explores only the effect of NCMS participation on health and does not indicate specific influential aspects of NCMS. The supplemental information provided in the literature review is insufficient; thus, future studies can investigate the effects of specific NCMS features on the health of the rural elderly. ***Third,*** this study explores the health changes in the rural elderly over the three-year implementation period of NCMS. This time span is exploratory and can be lengthened to investigate the sustained health concerns of the rural elderly. In addition, the medical security system in the rural areas of various countries can be compared with the NCMS to determine differences in their health effects.

## Conclusion

This study uses data of 2005 and 2008 obtained from the CLHLS database to explore the effect mechanism of NCMS on three domains of the health of the rural elderly. In the three years since the implementation of NCMS, the coverage of medical care service in rural areas rose. NCMS participation enhances the general health of the rural elderly and promotes their physical function. NCMS organized by Chinese government can also effectively ease fear, anxiety, and loneliness in the rural elderly; however, it increases the sense of uselessness. Based on these findings and our analysis, we present several suggestions: (1) consolidate the promotion effects of NCMS on general health and physical health to improve the system of primary health care; (2) consider psychological factors in medical assistance and support for the rural elderly; (3) strengthen knowledge propaganda; (4) strengthen the awareness of the elderly, that is, it is more important and effective to build a healthy body by physical exercises rather than rely on the medical care after falling sick Future studies can continuously focus on the health of the rural elderly.

## Competing interests

The authors declare that they have no competing interests.

## Authors’ contributions

YL wrote and revised the manuscript, was responsible for the design of the study, and performed the statistical analysis. PL participated in the design of the study. Both authors read and approved the final manuscript.
